# Wheeler graphs: A framework for BWT-based data structures^☆^

**DOI:** 10.1016/j.tcs.2017.06.016

**Published:** 2017-10-25

**Authors:** Travis Gagie, Giovanni Manzini, Jouni Sirén

**Affiliations:** a Diego Portales University and CEBIB, Santiago, Chile; b University of Eastern Piedmont, Alessandria, Italy; c IIT-CNR, Pisa, Italy; d Wellcome Trust Sanger Institute, Cambridge, UK

**Keywords:** Compressed data structures, Burrows–Wheeler transform, Pattern matching

## Abstract

The famous Burrows–Wheeler Transform (BWT) was originally defined for a single string but variations have been developed for sets of strings, labeled trees, de Bruijn graphs, etc. In this paper we propose a framework that includes many of these variations and that we hope will simplify the search for more.

We first define *Wheeler graphs* and show they have a property we call *path coherence*. We show that if the state diagram of a finite-state automaton is a Wheeler graph then, by its path coherence, we can order the nodes such that, for any string, the nodes reachable from the initial state or states by processing that string are consecutive. This means that even if the automaton is non-deterministic, we can still store it compactly and process strings with it quickly.

We then rederive several variations of the BWT by designing straightforward finite-state automata for the relevant problems and showing that their state diagrams are Wheeler graphs.

## 1 Introduction

The Burrows–Wheeler Transformation (BWT) has a very peculiar history. First conceived in 1983, it was published only eleven years later in a technical report [9], presumably because it was so innovative that the first reviewers were not able to grasp its full significance. A few years later, the compression algorithm bzip2 based on the BWT became popular, challenging gzip's dominance, thanks to the finely engineered implementation of Julian Seward [46] (the very same computer scientist who gave us also the invaluable tool Valgrind [47]).

After its introduction as a compression tool, interest in the BWT was rekindled when many researchers realized that, among the different techniques discovered at the turn of the century for designing compressed indexes [19], [29], [34], those based on the BWT are probably the simplest and most space efficient [15], [43]. After this realization, in the last ten years we have witnessed an unusual phenomenon in computer science: variants of the BWT have been proposed and applied to more and more complex objects: from trees, to graphs, to alignments. These variants are clearly related to the BWT even if some of them no longer have the two main features of the original BWT, namely of being invertible and of “helping” compression.

At this point it is natural to ask whether we have approached the BWT as the blind men approached the elephant (see, e.g., [45]), with one touching a leg and thinking the elephant is like a tree, another touching the trunk and thinking it is like a snake, and yet another touching the tail and thinking it is like a rope. We do not disparage previous surveys of the BWT and related data structures, such as [1], [35], [42], since we too have spent years trying to make sense of our sometimes disparate impressions of the it. Without pretending to give a complete answer, in this paper we propose a unifying view for many different BWT variants. Somewhat surprisingly we get our unifying view considering the Nondeterministic Finite Automata related to different pattern matching problems. We show that the state graphs associated to these automata have common properties that we summarize with the concept of Wheeler graphs.^1^

Using the notion of a Wheeler graph, we show that it is possible to process strings efficiently, e.g., in linear time if the alphabet is constant, even if the automaton is nondeterministic. In addition, we show that Wheeler graphs can be compactly represented and traversed using up to three arrays with additional data structures supporting efficient rank and select operations. It turns out that these arrays coincide with, or are substantially equivalent to, the output of many BWT variants described in the literature.

We believe our unifying view can help researchers develop new BWT variants and new indexing data structures. However, we stress that not every BWT-related data structure fits our framework: for example Ganguly et al.'s parameterized BWT [25] and the index for order-preserving matching in [23]. Therefore, we hope that our contribution will spur further research resulting in a wider vision of the fascinating field originated by the seminal work of Burrows and Wheeler.

## 2 Definitions and basic results

Consider a directed edge-labeled graph *G* such that each edge is labeled by a character from an totally-ordered alphabet *A*. We use ≺ to denote the ordering among *A*'s elements. Labels on the edges leaving a given node are not necessarily distinct, and there can be multiple edges linking the same pair of nodes (for simplicity we still use the term *graph* rather than the more formally correct *multi-graph*).

Definition 1 *G* is a *Wheeler graph* if there is an ordering of the nodes such that nodes with in-degree 0 precede those with positive in-degree and, for any pair of edges e=(u,v) and $e^{\prime }=(u^{\prime },v^{\prime })$ labeled *a* and $a^{\prime }$ respectively, the following monotonicity properties hold:

(1) $$\begin{array}{ccc} a≺a^{\prime }\; & ⟹\; & v<v^{\prime }\;,\\ (a=a^{\prime })\wedge (u<u^{\prime })\; & ⟹\; & v\leq v^{\prime }\;. \end{array}$$

**Fig. 1:**
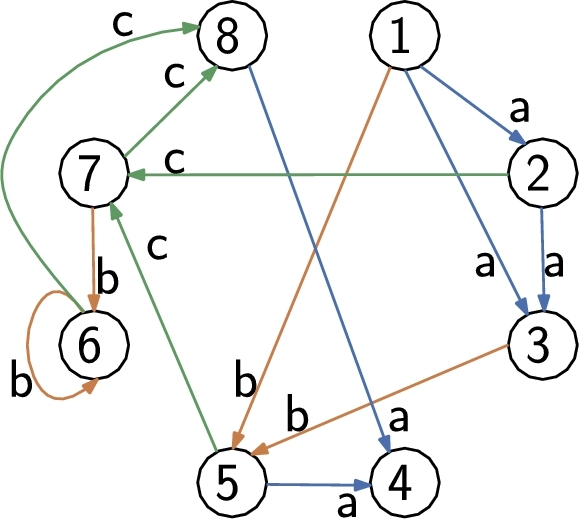
An eight-node Wheeler graph. Node 1 has in-degree 0; edges labeled a enters in nodes 2, 3, 4; edges labeled b in nodes 5, 6; edges labeled c in nodes 7, 8.

An example of a Wheeler graph is shown in Fig. 1. As an immediate consequence of (1), all edges entering a given node must have the same label. We now show that Wheeler graphs also possess the following property:

Definition 2 *G* is *path coherent* if there is a total order of the nodes such that for any consecutive range [i,j] of nodes and string *α*, the nodes reachable from those in [i,j] in |α| steps by following edges whose labels for *α* when concatenated, themselves form a consecutive range.

Lemma 3 *If G is a Wheeler graph by an ordering π on its nodes then it is path coherent by π.*

Proof Suppose *G* is a Wheeler graph by *π*. Consider a consecutive range [i,j] of nodes and let $\left[i^{\prime },j^{\prime }\right]$ be the smallest range that contains all the nodes reachable from those in [i,j] in one step by following edges labeled with some character *a*. By our choice of $\left[i^{\prime },j^{\prime }\right]$, both $i^{\prime }$ and $j^{\prime }$ are reachable from nodes in [i,j] in one step by following edges labeled *a*. By our definition of a Wheeler graph, nodes with in-degree 0 precede those with positive in-degree, such as $i^{\prime }$, so every node in $\left[i^{\prime },j^{\prime }\right]$ has at least one incoming edge.

Assume some node *v* strictly between $i^{\prime }$ and $j^{\prime }$ has an incoming edge labeled $a^{\prime }\neq a$. Since $i^{\prime }<v$ we have $a≺a^{\prime }$, by our definition of a Wheeler graph and *modus tollens*; similarly, since $v<j^{\prime }$ we have $a^{\prime }≺a$, thus obtaining a contradiction. It follows that the edges arriving at nodes in $\left[i^{\prime },j^{\prime }\right]$ are all labeled *a*. Furthermore, since the labels are equal, by the second implication in (1) and *modus tollens* we get that any edge with destination strictly between $i^{\prime }$ and $j^{\prime }$ must originate in [i,j].

It follows that the nodes reachable in one step from those in [i,j] by following edges labeled *a* are the ones in $\left[i^{\prime },j^{\prime }\right]$, which is a consecutive range. For any string *α*, therefore, the nodes reachable in |α| steps from those in [i,j] by following edges whose labels form *α*, themselves form a consecutive range, by induction on the length of *α*. □

In the later sections of this paper, we explore the implications of path coherence. In the remainder of this section we first show it is possible to obtain a fast and compact representation of a Wheeler graph, then sketch why Wheeler graphs can achieve compression. We point out that, even without explicitly defining Wheeler graph s, many researchers have implicitly considered them while studying how to implement efficiently BWT variants and how to bound their space usage, and given results analogous to the ones we give now.

A plain, edge-by-edge representation of a labeled graph with *n* nodes and *e* edges uses Θ(e(log⁡n+log⁡|A|)) bits. Given a Wheeler graph *G*, let $x_{1}<x_{2}<\cdots <x_{n}$ denote the ordered set of nodes. For i=1,…,n let $\ell_{i}$ and $k_{i}$ denote respectively the out-degree and in-degree of node $x_{i}$. Define the binary arrays of length e+n

(2) $$O=0^{\ell_{1}}1\;0^{\ell_{2}}1\cdots 0^{\ell_{n}}1,\;I=0^{k_{1}}1\;0^{k_{2}}1\cdots 0^{k_{n}}1.$$

Note that *O* (resp. *I*) consists of the concatenated unary representations of the out-degrees (resp. in-degrees). Let $L_{i}$ denote the multiset of labels on the edges exiting from $x_{i}$ arranged in an arbitrary order, and let L[1..e] denote the concatenation $L=L_{1}L_{2}\cdots L_{n}$. By construction, $|L_{i}|=\ell_{i}$ and there is a natural one-to-one correspondence between the 0's in *O* and the characters in *L*. For example, for the graph of Fig. 1 it is

$$\begin{array}{cccccccccc} O\; & \;\;=\;\;\; & 0001\; & 001\; & 01\; & 1\; & 001\; & 001\; & 001\; & 01\;\\ L\; & =\; & \mathrm{aab}\; & \mathrm{ac}\; & b\; & \; & \mathrm{ac}\; & \mathrm{bc}\; & \mathrm{bc}\; & a\; \end{array}$$

while the indegree array is I=101001001001001001001. Finally, let C[1..|A|] denote the array such that C[c] is the number of edges with label smaller than *c*. For simplicity, assume every distinct character labels some edge; otherwise, we store a bitvector of |A| bits marking the characters that do label edges and work with that subset. With this assumption, C[c]<C[c+1] and $x_{j}$ has all incoming edges labeled *c* if and only if $C[c]<\sum_{i\leq j}k_{i}\leq C[c+1]$.

Given an array *Z* we use the standard notation ${\mathrm{rank}}_{c}(Z,i)$ to denote the number of occurrences of *c* in Z[1,i], and ${\text{select}}_{c}(Z,j)$ to denote the position of the *j*-th *c* in *Z*. For simplicity we assume ${\mathrm{rank}}_{c}(Z,0)=0$. The following two properties are straightforward to prove; indeed, similar properties have been established in many papers dealing with BWT-related data structures.

1. The out-degree $\ell_{i}$ of node $x_{i}$ is ${\text{select}}_{1}(O,i)-{\text{select}}_{1}(O,i-1)$. The labels in the edges leaving $x_{i}$ are $L[w_{i}-\ell_{i}+1,w_{i}]$ where $w_{i}={\text{select}}_{1}(O,i)-i$.
2. If node $x_{i}$ has one or more outgoing edges labeled *c*, then the largest index *j* such that $\left(x_{i},x_{j}\right)$ has label *c* can be computed as follows. Let $w_{i}$ be defined as above and let $h_{i}={\mathrm{rank}}_{c}(L,w_{i})$. If we order edges by label with ties broken by origin, $\left(x_{i},x_{j}\right)$ is the $h_{i}$-th edge labeled *c*. Since there are C[c] edges with a label smaller than *c*, we have $j=1+{\mathrm{rank}}_{1}(I,{\text{select}}_{0}(I,h_{i}+C[c]))$. To find the smaller indices *j* such that $\left(x_{i},x_{j}\right)$ has label *c*, we decrement $h_{i}$ and repeat this procedure, until $h_{i}={\mathrm{rank}}_{c}(L,w_{i}-\ell_{i})+1$.

With a symmetric reasoning, given $x_{j}$ and *c* we can establish whether there exists an edge labeled *c* entering in $x_{j}$, and, if this is the case, all indices *i* such that $\left(x_{i},x_{j}\right)$ has label *c*. Using standard data structures to represent compactly arrays supporting rank and select queries [43], we can establish the following result.

Lemma 4 *It is possible to represent an n-node, e-edge Wheeler graph with labels over the alphabet A in* 2(e+n)+elog⁡|A|+|A|log⁡e+o(n+elog⁡|A|) *bits. The representation supports the forward and backward traversing of the edges in* O(log⁡|A|) *time.* □

We can often reduce even further the space used to represent Wheeler graphs. For example, suppose *G* is a Wheeler graph; $V_{k}$ is the set of nodes of *G* reachable in exactly *k* steps from some other nodes; *S* is a set of strings of length *k* such that every node in $V_{k}$ is reachable in *k* steps by following edges whose labels when concatenated form a string in *S*; and *f* is a function assigning to each node $v\in V_{k}$ a string s∈S such that *v* is reachable in *k* steps by following edges whose labels when concatenated form *s*. Consider the list $L_{k}$ of labels of edges originating in $V_{k}$ in order by origin with ties broken by label.

By Lemma 3, for s∈S all the nodes $v\in V_{k}$ such that f(v)=s form a consecutive interval. Therefore, we can partition $L_{k}$ into |S| consecutive intervals such that each interval $L_{s}$ is the concatenation of the labels of edges leaving nodes *v* such that f(v)=s; $L_{s}$ is empty for s∉S. It follows that if $k\leq (1-\epsilon ){\mathrm{lg}}_{\sigma }|V_{k}|$, where ϵ>0 is a constant and σ=|A| is the size of the alphabet of labels, then we can store $L_{k}$ in $\sum_{s\in A^{k}}|L_{s}|H_{0}(L_{s})+o(|V_{k}|)$ bits, where $H_{0}(L_{s})$ is the 0th-order empirical entropy of $L_{s}$. Informally, this means that if knowing how we can get to a node in *k* steps tells us a lot about the labels that are likely to be on the edges leaving that node, then we can compress $L_{k}$ well. In some ways this generalizes the well-known analyses [14], [39] of the compression achievable using the Burrows–Wheeler Transform on a single string.

## 3 NFAs, Wheeler graphs and FM-indexes

**Fig. 2:**
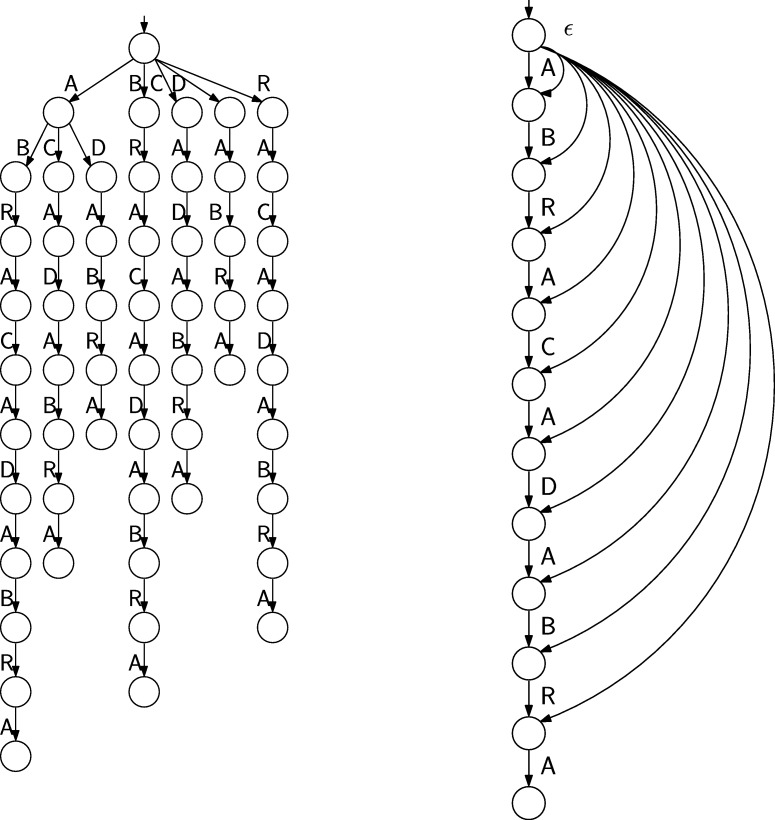
Deterministic (left) and non-deterministic (right) finite automata accepting all substrings of ABRACADABRA. All states are accepting.

Suppose we have never heard the words “Suffix Array” or “Burrows–Wheeler Transform” but we want to build a data structure supporting efficient queries for the substrings of, say, s=ABRACADABRA. A simple solution would be to build the Deterministic Finite Automaton (DFA) accepting all substrings of *s*, see Fig. 2 (left). Although there are techniques to compactly represent DFAs, such avenue will likely lead to a data structure much larger than the O(nlog⁡|A|) bits of the original text.

A more space economical alternative is to build the Nondeterministic Finite Automaton (NFA) for the same set of substrings: It has a linear structure, see Fig. 2 (right), but because of nondeterminism, processing of patterns appears to be a difficult task. It turns out that this difficulty is only a matter of perspective: traversing the DFA becomes much simpler if we recognize that it is a Wheeler graph in disguise.

**Fig. 3:**
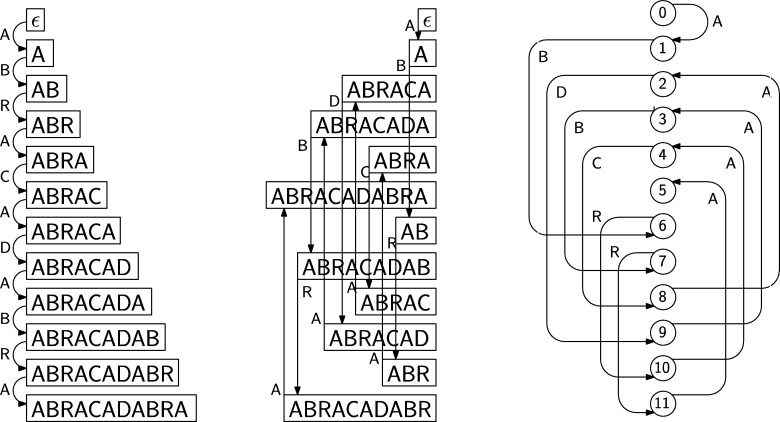
From the NFA (left) to the Wheeler graph (center) to the FM-index (right). All states are initial and accepting and numbered on the right to show that the diagram is a Wheeler graph.

The NFA of Fig. 2 is already a labeled directed graph. To make it a Wheeler graph we only need to eliminate the *ϵ*-transitions (which violate the property that all the incoming edges to a node have the same label), make all the states initial (so the language is not changed), and define an ordering of the nodes such that the monotonicity properties (1) hold. To this end, we associate to each node of the NFA the prefix of *s* that takes us there without an *ϵ*-transition and order the NFA nodes according to the right-to-left lexicographic rank of such strings, see Fig. 3 (left and center). With this definition the NFA without *ϵ*-transitions is a Wheeler graph. Once the node ordering is established we can discard the prefixes and identify each node with its rank in the ordering, see Fig. 3 (right).

In the Wheeler graph derived from the NFA of Fig. 3 every node has out-degree 1 except from node 5, corresponding to the longest prefix, that has out-degree zero. Hence, in the representation of the Wheeler graph described in Sect. 2, we can get rid of the bitarray *O* and instead insert in position 5 of *L* a symbol not occurring elsewhere in *s*, say $. Since all nodes have in-degree 1 except node 0 (ignoring the sourceless edges indicating that all nodes are initial) there is no need to store the bitarray *I* either. Summing up, we can represent and navigate the Wheeler graph using the string L=ABDBC$RRAAAA, enriched with data structures for rank/select operations, and the array C[1..|A|]. Since the string *L* coincides with the last column of the BWT matrix of $s^{R}$, the string *s* reversed, and *C* is a well-known representation of the first column of such matrix, we have established the following result.

Lemma 5 *Given a string s, let* NFA(s) *denote the corresponding NFA described above. The FM-index of* $s^{R}$ *is a compact representation of* NFA(s)*. The Last-to-First and First-to-Last maps of the FM-index coincides with the navigation operations in* NFA(s)*.* □

The attentive reader may ask why the Wheeler graph represents the FM-index of $s^{R}$ while historically the FM-index was defined for *s*. The reason is that the FM-index was derived from the BWT of *s*. The consequence is that the search of the pattern must be done right-to-left: the infamous backward-search procedure [20]. Here, we have defined the NFA so that the search is done left-to-right and through the Wheeler graph we have therefore obtained the FM-index of $s^{R}$.

Lemma 5 provides a new perspective on FM-indexes that may be interesting in its own right and, more importantly, suggests a way we can systematically generalize the ideas behind FM-indexes to indexing other kinds of data than single strings. Using the concept of path coherence, we now prove a more powerful version of this lemma:

Theorem 6 *Consider a finite-state automaton over an alphabet A without ϵ-transitions and with either one initial state or all states initial. If its state diagram is a Wheeler graph with n nodes and e edges then we can store it in* 2(e+n)+elog⁡|A|+|A|log⁡e+o(n+elog⁡|A|) *bits such that, for any string α, in* O(|α|log⁡|A|) *time we can compute the set of states reachable from the initial states on α.*

Proof Suppose the state diagram is a Wheeler graph by an ordering *π* on its nodes so, by Lemma 3, it is path coherent by *π*. Since either one state is initial or all are, the initial states form a consecutive range in *π*. By Definition 2, the nodes reachable from the initial states on each prefix of *α* form a consecutive interval. We can use Lemma 4 to map from the interval for each prefix to the interval for the next prefix in O(log⁡|A|) time. □

Theorem 6 means that if we have a problem we can solve with a finite-state automaton, then even if the automaton is non-deterministic we may still be able to implement it without the usual blowups in space or query time. In the rest of this paper we show that many data structures based on variants of the BWT fit into this framework.

We note as an aside that FM-indexes usually include a sampled suffix array which permits us to locate each occurrence of a pattern in the indexed string. Extending this idea to automata seems straightforward, by sampling the nodes, but we do not explore that in this paper.

## 4 Other Wheeler graphs in disguise

### 4.1 Multi-string BWT and permuterm index

The first natural generalization of the BWT is to extend it to a collection of strings. This was done for the first time in [36], [37], [38] where the authors described a reversible multi-string transformation inspired by the BWT, and showed its effectiveness for data compression and for measuring string similarity.

**Fig. 4:**
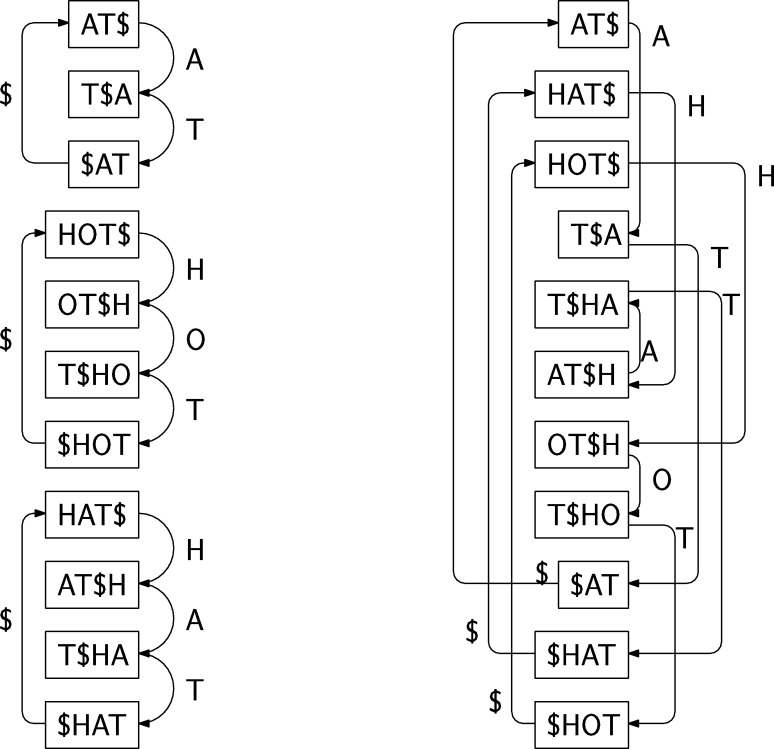
Left: A finite automaton accepting all circular substrings of AT$, HOT$, HAT$; all states are initial and accepting. Right: Sorting the states according to the associated circular shifts gives us a Wheeler graph.

In the context of pattern matching, given a collection of strings $s_{1},\ldots ,s_{d}$, in addition to substring queries it is convenient to offer also the possibility of *prefix/suffix* queries. In a prefix/suffix query, given two substring α,β we want to find all strings $s_{i}$ such that $s_{i}$ is prefixed by *α* and suffixed by *β*. As first observed in [21], [22] with the Compressed Permuterm Index, such queries can be solved by adding a special symbol $ to each string and searching for the pattern β$α inside a *circular* version of each $s_{i}\$$. Fig. 4 (left) shows the NFA supporting circular queries for the strings AT$, HOT$, HAT$. To each node we naturally associate a circular shift of one of the strings; if we sort the nodes according to the right-to-left lexicographic order of such shifts the resulting graph is a Wheeler graph, see Fig. 4 (right).

Since each node in the Wheeler graph has in-degree and out-degree 1, we can represent it with the approach of Lemma 4, using only the arrays *L* and *C*. Note that the array *L*, L=AHHTTAOT$$$ in the example of Fig. 4, coincides with the multi-string BWT defined in terms of the cyclic shifts in [38]. Instead, the Compressed Permuterm Index is built computing the single-string BWT of the concatenation $s_{1}\$s_{2}\cdots \$s_{d}$ that does not support naturally the search for circular patterns. The authors of [21] got around this problem by sorting the strings $s_{1},\ldots ,s_{d}$ before the concatenation and by applying the so-called jump2end function. More recent algorithms using circular pattern search all make use of the cyclic multi-string BWT [2], [7], [8], [30].

### 4.2 XBWT and trie representation

**Fig. 5:**
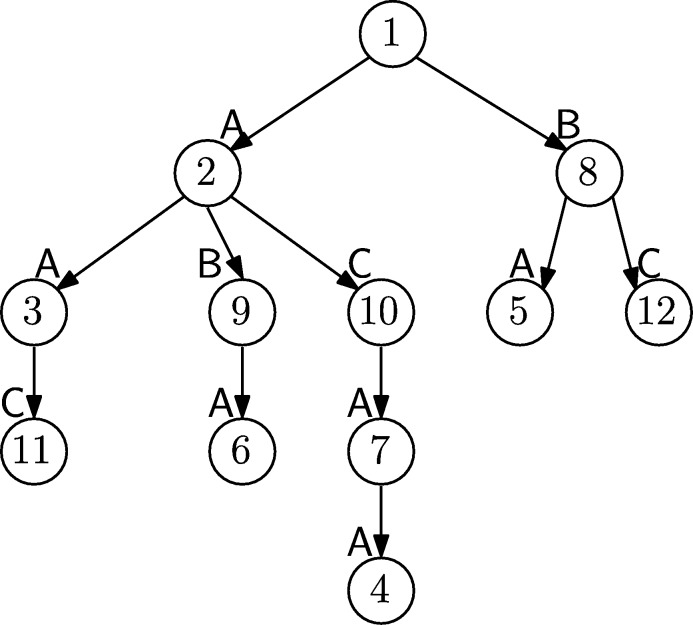
A finite automaton accepting all substrings of AAC, ABA, ACAA, BA and BC. All states are initial and accepting and numbered to show that the diagram is a Wheeler graph.

If we are only interested in the substrings of a collection $s_{1},\ldots ,s_{d}$, and not in circular matches, a smaller automaton than the one considered in the previous section is the one derived from the *trie* data structure, see Fig. 5. Following the lead from [16], [17], [18], we associate to each node the string formed by the labels in the node-to-root upward path, and we order all nodes according to the lexicographic rank of such strings.

The resulting graph is a Wheeler graph with the same number of nodes as the original trie. In a trie all nodes except the root have in-degree 1 so in the representation of Lemma 4 we do not need to store the in-degree binary array. Hence the representation consists of the out-degree binary array *O*, the labels array *L* and the count array *C*. For the trie in Fig. 5 we have

$$\begin{array}{ccccccccccccc} O\; & \;\;=\;\;\; & 001\; & 0001\; & 01\; & 1\; & 1\; & 1\; & 01\; & 001\; & 01\; & 01\; & 1\\ L\; & =\; & \mathrm{AB}\; & \mathrm{ABC}\; & C\; & \; & \; & \; & A\; & \mathrm{AC}\; & A\; & A\; & \end{array}$$

This representation is essentially equivalent to the XBWT (eXtended BWT) introduced in [16] to represent labeled trees, with the arrays *O* and *L* corresponding respectively to $S_{\mathrm{last}}$ and $S_{\alpha }$ in [16]. Note that we can restrict the search to prefixes by setting the root as the only start state, and we can restrict the search to suffixes by setting as accepting states only those corresponding to one of the input strings without affecting our representation.

### 4.3 de Bruijn graphs

Bowe, Onodera, Sadakane and Shibuya [6] (see also, e.g., [5], [10], [33]) extended the XBWT from trees to de Bruijn graphs, which are widely used in bioinformatics for de novo assembly, read correction, identifying genetic variations in a population, and other applications. A *k*th-order de Bruijn graph for a string or set of strings contains a node for each distinct *k*-tuple that occurs in those strings, and an edge (u,v) if there is a (k+1)-tuple in the strings that starts with *u* and ends with *v* (which implies that *v* can be obtained from *u* by deleting *u*'s first character and appending a character).

Together with the GCSA, described in Section 4.5, Bowe et al.'s representation is really the prototypical BWT-based data structure that requires us to think in terms of graphs instead of strings. The main difference between representing a labeled tree and representing a de Bruijn graph is that, in a graph, nodes can have in-degree more than 1, but this can be handled using the in-degree array *I* as described in Lemma 4.

**Fig. 6:**
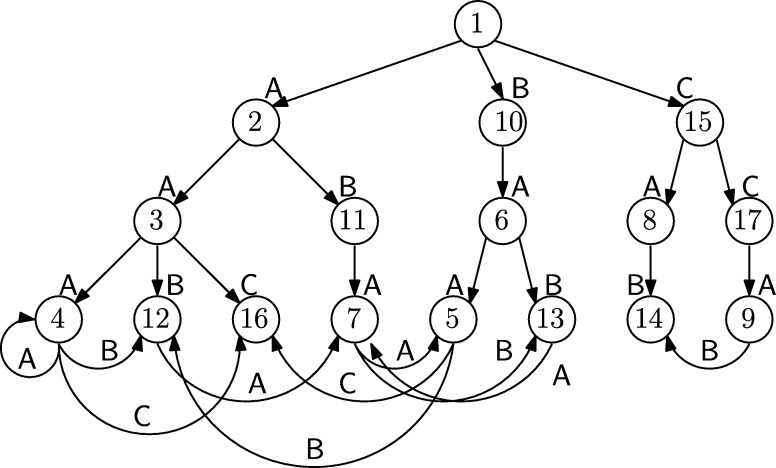
A finite automaton accepting all prefixes of length at least 3 of strings in which the only triples are AAA, AAB, AAC, ABA, BAA, BAB, CAB and CCA and in which BAAA and CABA do not occur. State 1 is initial, all states at distance 3 from the initial state are accepting. States are numbered to show that the diagram is a Wheeler graph.

We can also rederive Bowe et al.'s representation from Theorem 6: we build a finite-state automaton that accepts all prefixes of length at least *k* of strings containing only *k*-tuples and (k+1)-tuples from a given list, by building a trie for the *k*-tuples and then adding edges connecting the leaves appropriately. Fig. 6 shows an example for the triples AAA, AAB, AAC, ABA, BAA, BAB, CAB and CCA, with the edges for BAAA and CABA missing: this automaton accepts all prefixes of length at least 3 of strings in which the only triples are AAA, AAB, AAC, ABA, BAA, BAB, CAB and CCA and in which BAAA and CABA do not occur. By numbering each node according to the lexicographic rank of the string labeling the node-to-root path, the state diagram is immediately seen to be a Wheeler graph.

### 4.4 FM-index of alignment

Let *G* be a Wheeler graph under node ordering *π*. If all incoming edges to nodes in range [i,j] have the same label, we can merge the range into a single node *v*, and the resulting graph $G^{\prime }$ will still be a Wheeler graph. If graph *G* has an edge with label *a* from node *u* to a node in range [i,j], graph $G^{\prime }$ will have an edge (u,v) with label *a*. Similarly, if graph *G* has an edge with label $a^{\prime }$ from a node in range [i,j] to node *w*, graph $G^{\prime }$ will have an edge (v,w) with label $a^{\prime }$.

If we have a multi-string BWT, we can transform its Wheeler graph into a more compact representation of the strings by merging ranges of nodes. This compact representation may contain *false positives*: path labels that combine substrings from different original strings. The *FM-index of alignment* (FMA) [40], [41] has an efficient procedure for detecting false positives, based on carefully choosing the nodes to merge.

**Fig. 7:**
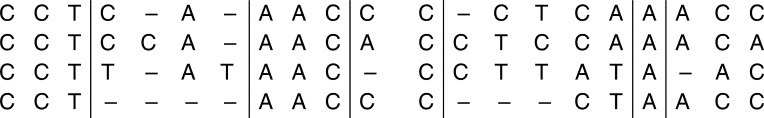
Left: An alignment of strings CCTCAAACC, CCTCCAAACA, CCTTATAAC, and CCTAACC. The alignment has been separated into common and non-common regions. Right: The alignment transformed for FMA. The suffixes moved to the non-common regions are CT for the first common region and AC for the second one.

We start with an alignment of the strings, and separate the alignment into *common* regions $X_{i}$ shared between all strings, and *non-common* regions $Y_{i}$ where some strings are different. We assume that each common region has a proper suffix $Z_{i}$ that does not occur anywhere else in the strings. If no such suffix exists, we merge the common region into the neighboring non-common regions. We transform the alignment in two steps. First we move the suffixes $Z_{i}$ into the following non-common regions $Y_{i}$. Then we justify each non-common region to the right, and use $X_{i}^{\prime }$ and $Y_{i}^{\prime }$ to denote the common and non-common regions in the transformed alignment. See Fig. 7 for an example.

**Fig. 8:**
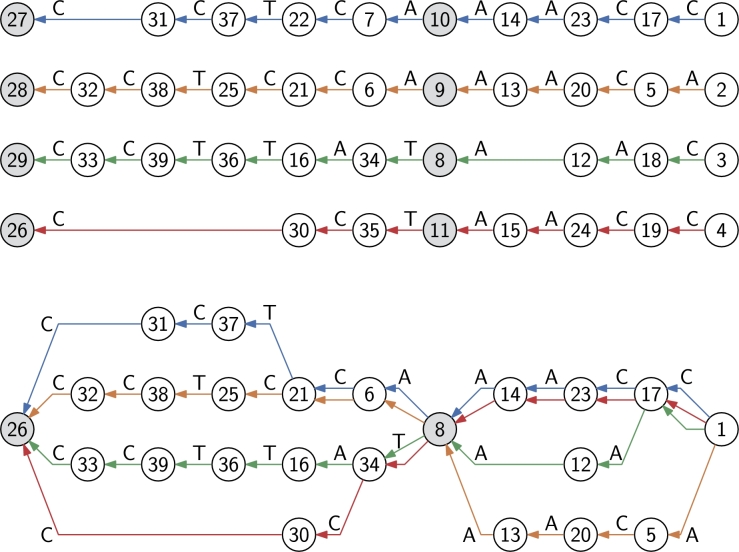
Top: Wheeler graph of the multi-string BWT of the transformed alignment in Fig. 7. Node ordering is based on the labels in the nodes. Gray nodes correspond to the common regions. Bottom: Wheeler graph of the FMA for the same alignment. The colors of each edge mark the original strings that cross the edge. The labels of non-merged nodes are the same as in the top graph for convenience. (For interpretation of the colors in this figure, the reader is referred to the web version of this article.)

The transformed alignment guides us in merging the Wheeler graph of the multi-string BWT into the FM-index of alignment. As we are building an FM-index for the original strings, we need to build a Wheeler graph for the reverse strings. We merge nodes corresponding to aligned positions, if the nodes a) have in-degree 0; b) are in a common region; or c) correspond to the same suffix of the non-common region. See Fig. 8 for an example.

By Lemma 3, the Wheeler graph of a multi-string BWT is path coherent. To see that the graph remains a Wheeler graph after merging, we note that:

1. Source nodes with in-degree 0 form a consecutive range by Definition 1.
2. A common region $X_{i}^{\prime }$ is entered either from the source nodes or from the following non-common region $Y_{i}^{\prime }$. In the latter case, the set of nodes reachable with string $Z_{i}^{R}$ is the set of nodes with outgoing edges to the common region $X_{i}^{\prime }$. By Lemma 3, these nodes form a consecutive range. As the common region is entered from a consecutive range of nodes, we see by iterating Definition 2 that nodes corresponding to aligned positions in the region also form consecutive ranges.
3. By the previous two cases, the non-common region is entered from a consecutive range of nodes. By Definition 2, we see that the nodes corresponding to a suffix of the region form consecutive ranges.

False positives are paths that do not correspond to any of the original strings. They can occur when the path comes from a non-common region $Y_{i+1}^{\prime }$, enters a common region $X_{i+1}^{\prime }$, and exits into another non-common region $Y_{i}^{\prime }$. Assume that for each node *v* we have stored the set of strings $S_{v}$ passing through the node. To check for false positives, we take the intersection of sets $S_{v}$ over all nodes *v* on the path. If the intersection is non-empty, it contains the strings *compatible* with the path. If (u,v) is the only outgoing edge from node *u*, we have $S_{u}\subseteq S_{v}$. Similarly, if (u,v) is the only incoming edge to node *v*, we have $S_{v}\subseteq S_{u}$. Hence it is enough to take the intersection a) at the final node; and b) at nodes *u*, where the path crosses an edge (u,v) to a node *v* with multiple incoming edges. It is also enough to store the set $S_{u}$ explicitly if a) node *u* has multiple or no outgoing edges; or b) $S_{u}\neq S_{v}$ for the only outgoing edge (u,v).

Let *α* be a string, let $V_{\alpha }$ be the set of nodes reachable by following paths with label *α*, and let $S_{\alpha }$ be the set of strings compatible with the paths. Because the graph is a DFA, the set of reachable nodes is monotonically decreasing: $\left|V_{\alpha a}\right|\leq \left|V_{\alpha }\right|$ and $\left|V_{a\alpha }\right|\leq \left|V_{\alpha }\right|$ for any character a∈A. If $Z_{i}$ is a moved substring, $\left|V_{Z_{i}^{R}a}\right|\leq 1$ for any character a∈A, as the paths can only end at the rightmost node of the common region $X_{i}^{\prime }$. If set $V_{Z_{i}^{R}a}$ is non-empty, set $S_{Z_{i}^{R}a}$ contains all strings, as $aZ_{i}$ is a substring of all strings.

When we search in FMA, we move from $V_{\alpha }$ to $V_{\alpha a}$. We update the set of compatible strings *S* lazily. Initially the set contains all strings. If $\left|V_{\alpha }\right|>1$, there cannot be false positives. Either none of the paths enters a common region from a non-common region, or all such paths start within $Z_{i}$ and enter $X_{i}^{\prime }$ through every incoming edge. In either case, $S_{\alpha }={⋃}_{v\in V_{\alpha }}S_{v}$. If $\left|V_{\alpha a}\right|=1$ and the node $v\in V_{\alpha a}$ has multiple incoming edges, there is a risk of false positives. We therefore update $S\leftarrow S\cap {⋃}_{v\in V_{\alpha }}S_{v}$. When the search finishes at $V_{\alpha^{\prime }}$, the set of compatible strings is $S\cap {⋃}_{v\in V_{\alpha^{\prime }}}S_{v}$.

### 4.5 GCSA

While not all NFAs have an ordering of nodes that makes them Wheeler graphs, we can use Wheeler graphs to index path labels of length up to *k* in arbitrary graphs [49]. This is the idea behind Generalized Compressed Suffix Arrays (GCSAs). We emphasize that Wheeler graphs can be larger than the graphs they are used to index.

Definition 7 Let *G* be an NFA, let $G^{\prime }$ be a Wheeler graph, let *α* be a string, and let $V_{\alpha }$ and $V_{\alpha }^{\prime }$ be the sets of nodes reachable with *α* in *G* and $G^{\prime }$, respectively. Graph $G^{\prime }$ is a *k*th-order *path graph* of *G* for a k>0, if there is a function *f* from nodes of $G^{\prime }$ to sets of nodes of *G* such that $V_{\alpha }={⋃}_{v\in V_{\alpha }^{\prime }}f(v)$ for all |α|≤k.

**Fig. 9:**
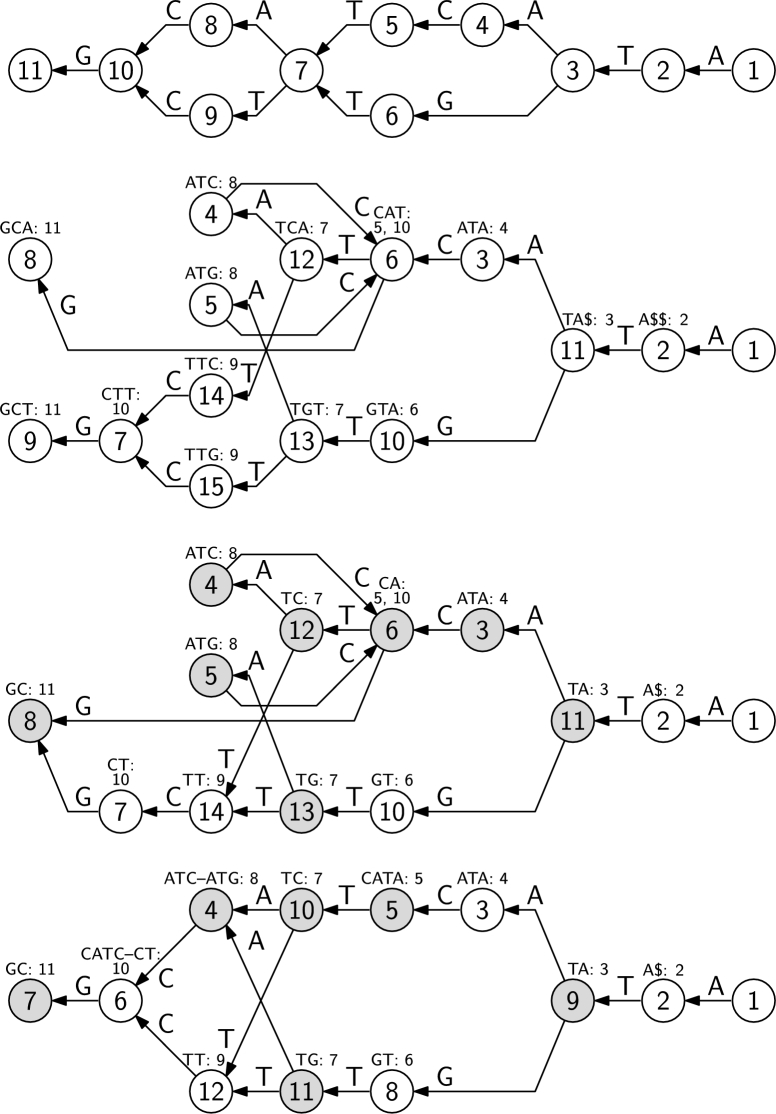
Top: A DFA. Second: A 3rd-order de Bruijn graph for path labels in the DFA. Node labels indicate node ordering in the Wheeler graph. We also show the *k*-tuples corresponding to each node (in reverse to match the sorting order) and the mapping *f* to the nodes of the DFA. Third: A pruned de Bruijn graph for the DFA. Node labels are compatible with the second graph. Gray color indicates nodes where the mapping must be stored explicitly. Bottom: A path graph based on a 4th-order de Bruijn graph.

We can use a *k*th-order de Bruijn graph of path labels in graph *G* as a *k*th-order path graph of *G*. See Fig. 9 for an example. Function *f* has the same role as the suffix array. As with the sets of strings in FMA, we must store set f(u) explicitly, if a) node *u* has multiple or no outgoing edges; or b) we cannot derive f(u) from f(v) for the only outgoing edge (u,v). We often number the nodes of the NFA in a way such that f(u)={x−1|x∈f(v)}, if (u,v) is the only outgoing edge from *u* and the only incoming edge to *v*.

With large values of *k*, de Bruijn graphs often have many redundant nodes when we use them as path graphs. If |α|≤k, the nodes $V_{\alpha }^{\prime }$ reachable with *α* in the de Bruijn graph are the ones with *α* as a suffix of the corresponding *k*-tuples. By Lemma 3, the nodes form a consecutive range. If $f(v)=f(v^{\prime })$ for all $v,v^{\prime }\in V_{\alpha }^{\prime }$, we can merge the nodes without affecting reachability. *GCSA2* [49] uses such *pruned de Bruijn graphs* to save space when indexing path labels in an NFA. See Fig. 9 for an example.

The original *GCSA* [50] considers a different scenario. Instead of indexing paths of length up to *k* in an arbitrary NFA, it indexes paths of arbitrary length in an acyclic DFA. Conceptually, GCSA construction searches for a de Bruijn graph $G^{\prime }$ that is equivalent to the input graph *G* as a DFA, and then uses that de Bruijn graph as an infinite-order path graph of *G*. Consecutive ranges of nodes are merged if $f(v)=f(v^{\prime })$ for all nodes *v* and $v^{\prime }$ in the range, even if the ranges do not correspond to shared suffixes of the *k*-tuples. See Fig. 9 (bottom) for an example.

### 4.6 PBWT, wavelet trees and wavelet matrices

Another remarkable variant of the BWT is Durbin's positional BWT [12] (PBWT), which he introduced for haplotype matching but has also been used for reconstructing ancestral recombination graphs [48]. Given *d* strings of equal length the PBWT supports the matching of substrings starting at a specified position in the strings.

We can view the PBWT as a Wheeler graph with the same structure as that of the multi-string BWT. If the multi-string BWT has an edge (u,v) with label *a* from text position *i* to position i+1, PBWT uses (i+1,a) as the label. Because all edges from position *i* go to position i+1, the positional component is always i+1 for all outgoing edges in range [(i−1)d+1,id]. Hence, we can infer the position from the range and store just the character *a* in the succinct representation (Lemma 4). A generalization [44] of PBWT relaxes the requirements. Instead of storing strings of equal length, we store the labels of paths in a graph. If edge (u,v) in the Wheeler graph corresponds to edge $\left(u^{\prime },v^{\prime }\right)$ with label *a* in the underlying graph, we use $\left(v^{\prime },a\right)$ as the label of the edge. As we can no longer infer the positional component, we have to store it explicitly.

Although introduced for completely different applications, the PBWT bears a striking resemblance to a data structure called a wavelet matrix, which Claude, Navarro and Ordóñez [11] introduced as a version of the wavelet tree [28] better suited for large alphabets. This suggests that even wavelet trees and matrices can be viewed as Wheeler graphs. For the sake of brevity, we assume the reader is familiar with these data structures with “myriad virtues” [13], [24], and note only that, while building a wavelet tree is analogous to a most-significant-bit-first radix sort, building either a PBWT or a wavelet matrix is analogous to a least-significant-bit-first radix sort.

**Fig. 10:**
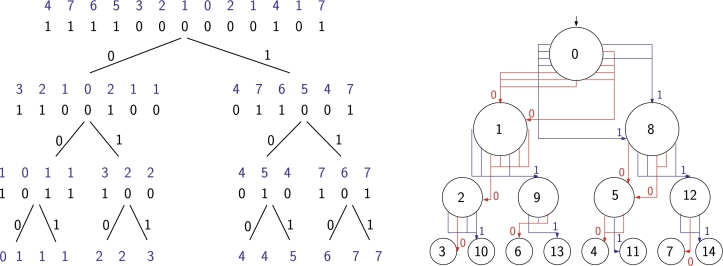
A standard representation of a wavelet tree and its representation as a Wheeler graph.

Consider the wavelet tree shown on the left in Fig. 10. Since it is a tree, we can use our construction from Subsection 4.2 to build an FSA (with one initial state) that accepts all the binary strings that are root-to-leaf paths in the wavelet tree. This does not store all the information in the wavelet tree, however, so we turn the graph from an FSA into a directed multi-graph, as shown on the right in Fig. 10, which is still a Wheeler graph. This graph has some interesting properties: e.g., the order of the outgoing edges from each node encode the bitvector stored at that node in the wavelet tree; as in the wavelet tree, for each internal node *v* except the root, *v*'s in-degree and out-degree are equal and its in-degree is equal to the sum of the in-degrees of its leaf descendants. It is beyond the scope of this paper to investigate this representation of wavelet trees as a data structure, but it seems like an interesting direction for future research that goes beyond the reach of Theorem 6.

**Fig. 11:**
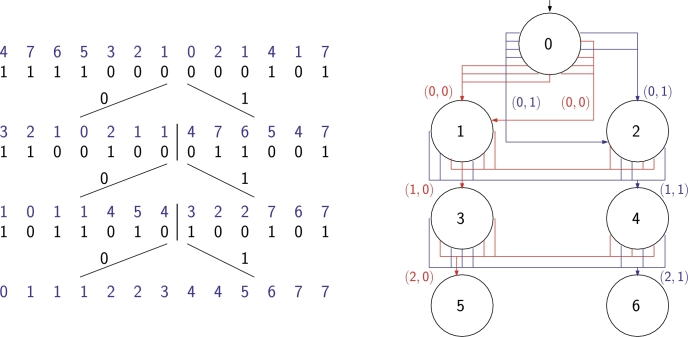
A standard representation of a wavelet matrix and its representation as a Wheeler graph with edge labels over a larger alphabet.

Now consider the wavelet matrix show on the left in Fig. 11. We can turn it into a Wheeler graph if, as in the wavelet tree, we transform it into a directed multi-graph and then, as in the PBWT, we rename the label *c* on each edge with the pair (i,c), where *i* is the level of the starting node, as shown on the right in Fig. 11. Again, the Wheeler graph clearly encodes the wavelet matrix, but we leave as future work investigating the possible benefits of this alternative representation.

## 5 Conclusions and future work

We have defined Wheeler graphs to try to capture the ideas behind many of the variants of the BWT, and given a framework for developing new variants by solving problems with finite-state automata whose state diagrams are Wheeler graphs. We did not pursue the topic here, but Wheeler graphs seem able to capture properties also of string transformations not related to pattern matching: by reversing the inequality $u<u^{\prime }$ in (1) we get a slightly different notion of Wheeler graph that can be used to succinctly represent the variant of the BWT defined in terms of the alternating lexicographic order [26] (see also [27] for an ancestor of the multi-string BWT).

There has been so much work involving the BWT, however, that it would be surprising if one idea could subsume it all, and indeed some BWT-related results, such as the positional BWT, seemingly cannot be reasonably modeled by finite-state automata, even if they can still be viewed as Wheeler graphs; other BWT-related results, such as Ganguly et al.'s parameterized BWT [25] and the index for order-preserving matching in [23], seem not even to be based on Wheeler graphs at all. We have not yet even considered bidirectional FM-indexes [3], [31], [32] and bidirectional BWT-based de Bruijn graphs [4].

Apart from applying and extending our framework, we hope to develop algorithms to recognize Wheeler graphs efficiently, and to characterize classes of finite-state diagrams that are Wheeler graphs or can be expanded slightly to become Wheeler graphs without changing the language accepted by the automata (although characterizing all such state diagrams might be difficult). In this regard we observe that not all regular languages have finite-state automata whose state diagrams are Wheeler graphs: e.g., in any finite-state automaton for the language $\left(ax^{⁎}b)|(cx^{⁎}d\right)$, there must be disjoint paths for $ax^{k}b$ and $cx^{k}d$ and both ends of all edges with label *x* must be in the same order, so there must be separate nodes for $x^{i}b$ and $x^{i}d$ for all values of *i*.

Finally, we hope our new perspective on BWT variants makes them more accessible to computer scientists from areas outside string algorithms and data structures.

## Footnotes

1 On many occasions Mike Burrows stated that, as reported also in [9], the original idea of the BWT is due to David Wheeler. We therefore decided to name this graph class after this pioneer of computer science.
